# Clinical characteristics and outcomes among critically ill patients with cancer and COVID-19-related acute respiratory failure

**DOI:** 10.1186/s12890-024-02850-z

**Published:** 2024-01-15

**Authors:** Ying-Ting Liao, Hsiao-Chin Shen, Jhong-Ru Huang, Chuan-Yen Sun, Hung-Jui Ko, Chih-Jung Chang, Yuh-Min Chen, Jia-Yih Feng, Wei-Chih Chen, Kuang-Yao Yang

**Affiliations:** 1 Department of Chest Medicine, Taipei Veterans General Hospital, Taipei, Taiwan; 2 School of Medicine, College of Medicine, National Yang Ming Chiao Tung University, Taipei, Taiwan; 3 Institute of Emergency and Critical Care Medicine, College of Medicine, National Yang Ming Chiao Tung University, Taipei, Taiwan; 4 Cancer Progression Research Center, National Yang Ming Chiao Tung University, Taipei, Taiwan; 5 Department of Medical Education, Taipei Veterans General Hospital, Taipei, Taiwan

**Keywords:** Acute respiratory failure, Coronavirus disease 2019 (COVID-19), Malignancy, Vasopressor, Inflammatory marker

## Abstract

**Background:**

Coronavirus disease 2019 (COVID-19) has affected individuals worldwide, and patients with cancer are particularly vulnerable to COVID-19-related severe illness, respiratory failure, and mortality. The relationship between COVID-19 and cancer remains a critical concern, and a comprehensive investigation of the factors affecting survival among patients with cancer who develop COVID-19-related respiratory failure is warranted. We aim to compare the characteristics and outcomes of COVID-19-related acute respiratory failure in patients with and without underlying cancer, while analyzing factors affecting in-hospital survival among cancer patients.

**Methods:**

We conducted a retrospective observational study at Taipei Veterans General Hospital in Taiwan from May to September 2022, a period during which the omicron variant of the severe acute respiratory syndrome coronavirus 2 was circulating. Eligible patients had COVID-19 and acute respiratory failure. Clinical data, demographic information, disease severity markers, treatment details, and outcomes were collected and analyzed.

**Results:**

Of the 215 enrolled critically ill patients with COVID-19, 65 had cancer. The patients with cancer were younger and had lower absolute lymphocyte counts, higher ferritin and lactate dehydrogenase (LDH) concentrations, and increased vasopressor use compared with those without cancer. The patients with cancer also received more COVID-19 specific treatments but had higher in-hospital mortality rate (61.5% vs 36%, *P =* 0.002) and longer viral shedding (13 vs 10 days, *P =* 0.007) than those without cancer did. Smoking [odds ratio (OR): 5.804, 95% confidence interval (CI): 1.847–39.746], elevated LDH (OR: 1.004, 95% CI: 1.001–1.012), vasopressor use (OR: 5.437, 95% CI: 1.202–24.593), and new renal replacement therapy (OR: 3.523, 95% CI: 1.203–61.108) were independent predictors of in-hospital mortality among patients with cancer and respiratory failure.

**Conclusion:**

Critically ill patients with cancer experiencing COVID-19-related acute respiratory failure present unique clinical features and worse clinical outcomes compared with those without cancer. Smoking, elevated LDH, vasopressor use, and new renal replacement therapy were risk factors for in-hospital mortality in these patients.

**Supplementary Information:**

The online version contains supplementary material available at 10.1186/s12890-024-02850-z.

## Background

The COVID-19 pandemic posed a considerable global challenge. The pandemic resulted in up to 6.9 million deaths (till November 2023, according to the data from World Health Organization) [1] and placed substantial burdens on health-care systems worldwide. Individuals with certain pre-existing conditions are particularly susceptible to COVID-19, and the relationship between COVID-19 and cancer has become a crucial and concerning issue. Patients with cancer are more susceptible to severe illness from COVID-19 than are those without cancer, which may be due to the presence of concurrent comorbidities, the inherent immunosuppressive characteristics of cancer, and the immunosuppression induced by systemic cancer treatments [2], with mortality rates as high as 25% being reported for patients with solid organ malignancies [3]. Respiratory failure is a severe complication of COVID-19 that typically occurs approximately 1 week after the onset of symptoms. Respiratory failure is usually accompanied by thrombosis and acute renal failure [4]. Treatment strategies for COVID-19-related respiratory failure are similar to those established for acute respiratory distress syndrome (ARDS) [5], and include oxygen therapy; lung-protective ventilation; prone positioning; supportive care; and administration of specific medications, such as corticosteroids, antiviral agents, immunomodulators, and anticoagulants [4–6]. Treatment for COVID-19-related respiratory failure among patients with cancer requires a multidisciplinary approach. The risk of death from COVID-19 among cancer patients is influenced by age; male sex; performance status; comorbidities; and hematological malignancies [7–10]. Whether recent cancer treatment influence survival remains controversial [2, 11, 12]. Understanding the factors that increase the risk of death from COVID-19 is crucial for optimizing patient management and improving outcomes. This study aims to investigate and compare the characteristics and outcomes among patients experiencing COVID-19-related acute respiratory failure between individuals with and without underlying cancer, while further analyzing the factors influencing in-hospital survival among cancer patients.

## Methods

This retrospective observational study was conducted at Taipei Veterans General Hospital, a tertiary medical center in Taiwan, between May and September 2022. During this period, the omicron variant of severe acute respiratory syndrome coronavirus 2 (SARS-CoV-2) was circulating in Taiwan. Patients were included in this study if they were infected with SARS-CoV-2 and experienced acute respiratory failure, defined as requiring high-flow nasal cannula (HFNC), or noninvasive ventilation (NIV), or mechanical ventilation (MV). SARS-CoV-2 infection was confirmed through reverse transcription polymerase chain reaction (RT-PCR) by using the Roche Cobas 6800 system (Roche Diagnostics, Rotkreuz, Switzerland).

Electronic medical records were reviewed to collect clinical information. Patients with advanced stage or metastatic cancer and those without remission were included. Other demographic data, including age, sex, body mass index (BMI), smoking and vaccination history, underlying diseases, do not resuscitate (DNR) code status, laboratory results on admission, and severity, were also obtained. Severity was assessed on the day of respiratory failure, including sequential organ failure assessment (SOFA) scores, Mean arterial pressure (MAP) scores (derived from the SOFA score, accounted for the administration of vasoactive agents, rating as 0 (no hypotension), 1 (mean arterial pressure < 70 mmHg), 2 (dopamine ≤5 mcg/kg/min or any dose of dobutamine), 3 (dopamine > 5 mcg/kg/min, epinephrine ≤0.1 mcg/kg/min, or norepinephrine ≤0.1 mcg/kg/min), and 4 (dopamine > 15 mcg/kg/min, epinephrine > 0.1 mcg/kg/min, or norepinephrine > 0.1 mcg/kg/min) [13], Acute Physiologic Assessment and Chronic Health Evaluation (APACHE) II score [14], Glasgow coma scale [15], vasopressor usage, PaO_2_/FiO_2_ ratio (estimated as the ratio of arterial oxygen partial pressure [PaO_2_ in mmHg] to fractional inspired oxygen) [16] were collected upon the day of respiratory failure. Treatment information, including receiving corticosteroids, tocilizumab, remdesivir, nirmatrelvir/ritonavir, molnupiravir, and enoxaparin; surgery; and new renal replacement therapy during admission, was also reviewed. Cytomegalovirus (CMV) infection, gastrointestinal bleeding, and thromboembolism were included as disease-related complications. Clinical courses and outcomes, such as the use of MV and ECMO, in-hospital mortality, and duration from the onset of symptoms until the day the cycle threshold (Ct) value exceeded 30, were also recorded [17]. Studies revealed a Ct value of 30 or higher to be non-infectious, with no virus isolated from culture [18]. In addition, a Ct value of at 30 or higher is the threshold for isolation release set by the Taiwan Center for Disease Control [19].

### Statistical analysis

The baseline characteristics were summarized using descriptive statistics, and continuous variables were expressed as medians and interquartile ranges. The Mann-Whitney U test was employed to assess differences in distribution between two independent groups for non-normally distributed continuous variables. Pearson’s chi-square test or Fisher’s exact test were used to examine variations in the distribution of categorical variables across different groups. In-hospital survival time and time to reach Ct > 30 among the patients with and without cancer were plotted using the Kaplan-Meier method and compared using a log-rank test. Cox proportional hazard models were used to assess the factors associated with in-hospital mortality, and factors with *P* < 0.1 in univariable analysis were incorporated into multivariable analysis. Statistical significance was indicated by *P* < 0.05. Statistical analyses were performed using IBM SPSS Statistics, version 26.0 (IBM, Armonk, NY, USA).

## Results

In total, 215 patients with COVID-19-related acute respiratory failure were enrolled. Among these patients, 65 had cancer. The patient characteristics, laboratory results, disease severity on the day of respiratory failure, treatment, complications, and outcomes are summarized in Table 1.

**Table 1:** Characteristics between COVID-19 patients with respiratory failure with and without cancer

	All cases (*n* = 215)	Cancer (*n* = 65)	No Cancer (*n* = 150)	*P* value***
**Demographics**				
Age, year, median	80	73	82	0.001
Male	145(67.4)	43(66.2)	102(68)	0.791
Body mass index, kg/m^2^, median	21.9	22.84	21.71	0.521
BMI^a^ < 18	36(18.3)	18.5(20.3)	24(16)	0.624
BMI > 24	62(31.5)	23(35.4)	39(26)	0.138
Vaccination doses, median	2	2	2	0.392
Ever vaccinated	143(66.5)	45(69.2)	98(65.3)	0.730
Fully vaccinated (> = 3 doses)	93(43.3)	32(49.2)	61(40.7)	0.244
Smoker	53(24.7)	20(30.8)	33(22)	0.171
DNR^a^	146(67.9)	47(72.3)	99(66)	0.363
**Comorbidity**				
Cerebrovascular disease	37(17.2)	6(9.2)	31(20.7)	0.041
Dementia	21(14.4)	5(7.7)	26(17.3)	0.065
Heart failure	22(10.2)	1(1.5)	21(14%)	0.003
Myocardial infarction	3(1.4)	0	3(2)	0.338
Peripheral vascular disease	11(5.1)	2(3.1)	9(6)	0.372
Diabetes mellitus	86(40)	22(33.8)	64(42.7)	0.225
Chronic kidney disease	50(23.3)	12(19.5)	34(24)	0.539
End stage renal disease	24(11.2)	5(7.7)	19(12.7)	0.287
Peptic ulcer	8(3.7)	0	8(5.3)	0.057
Hepatobiliary disease	21(9.8)	11(16.9)	10(6.7)	0.051
Chronic obstructive pulmonary disease	14(6.5)	4(6.2)	10(6.7)	0.889
Bronchiectasis	1(0.5)	0	1(0.7)	0.696
Interstitial lung disease	2(0.9)	1(1.5)	1(0.7)	0.516
Chronic oxygen use	9(4.2)	3(4.6)	6(4)	0.546
**Laboratory data on the day of respiratory failure (median)**				
White blood cells, 10^9^/L	11,150	10,060	11,640	0.800
Absolute lymphocyte count, 10^9^/L	708	546.8	781.6	0.003
Albumin, g/dL	3.05	3.1	3	0.795
C-reactive protein, mg/dL	6.1	6.89	5.91	0.331
Procalcitonin, ng/mL	0.72	0.68	0.75	0.909
Ferritin, ng/mL	668	1035	529	0.002
Lactic dehydrogenase, U/L	363	423	339	0.010
Lactate, mg/dL	23.3	26.4	23.15	0.274
D-dimer, ug/mL	2.472	2.35	2.62	0.600
Fibrinogen, mg/dL	381	378.1	390.7	0.453
Platelet count, /uL	182,000	159,000	186,500	0.090
**Severity on the day of respiratory failure**				
PaO2/FiO2 ratio, median	140	134.9	144	0.437
SOFA^a^ score, median	8	8	8	0.705
APACHE II^a^ score, median	24	24	24	0.612
MAP score^b^, median	1	1	0.5	0.022
Vasopressor use	70(32.6)	28(43.1)	42(28)	0.030
GCS^a^, median	7	8	7	0.286
**Treatment**				
Mechanical ventilation	131(60.9)	42(64.6)	89(59.3)	0.466
Surgery	60(27.9)	16(24.6)	44(29.3)	0.479
New renal replacement therapy during admission	21(9.8)	10(15.4)	11(7.3)	0.068
Extracorporeal membrane oxygenation	8(3.7)	5(7.7)	3(2)	0.056
Tocilizumab	76(35.3)	30(46.2)	46(30.7)	0.029
Remdesivir	169(78.6)	59(90.8)	110(73.3)	0.004
Nirmatrelvir/ritonavir	6(2.8)	4(6.2)	2(1.3)	0.117
Molnupiravir	11(5.1)	2(3.1)	9(6)	0.511
Enoxaparin	72(33.5)	22(33.8)	50(33.3)	0.942
Corticosteroid	184(85.6)	61(93.8)	123(82)	0.023
**Complications**				
CMV^a^ infection	38(17.7)	16(24.6)	22(14.7)	0.198
Gastrointestinal bleeding	62(28.8)	17(26.2)	45(30)	0.567
Thromboembolism	13(6.0)	7(10.8)	6(4)	0.084
**Outcomes**				
ICU^a^ admission	159(77.6)	48(73)	111(74)	0.987
Hospital length of stay, days, median	27	28	25	0.971
In-hospital mortality	94(43.7)	40(61.5)	54(36)	0.002
28 days mortality	69(30.7)	24(36.9)	42(28)	0.193
Time from symptoms onset to 1st Ct^a^ > 30, days, median	11	13	10	0.007

^a^BMI, Body mass index; DNR, Do not resuscitate; SOFA, Sequential Organ Failure Assessment; APACHE, Acute Physiology and Chronic Health Evaluation; MAP, Mean arterial pressure; GCS, Glasgow coma scale; CMV, Cytomegalovirus; ICU, Intensive Care Unit; Ct, cycle threshold

^b^MAP score is defined from the calculation of SOFA score, with inotropic doses as mcg/kg/min: 0, No hypotension; 1, MAP < 70 mmHg; 2, Dopamine ≤5 or Dobutamine (any dose); 3, Dopamine > 5, Epinephrine ≤0.1, or norepinephrine ≤0.1; 4, Dopamine > 15, Epinephrine > 0.1, or Norepinephrine > 0.1

*** Between patients with and without malignancy

The patients with cancer were younger than those without cancer (median age 73 vs 82 years, *P* = 0.001). Furthermore, the patients with cancer had lower prevalence rates of cerebrovascular accidents (9.2% vs 20.7%, *P* = 0.041) and heart failure (1.5% vs 14%, *P* = 0.003) than did the patients without cancer.

The patients with cancer had lower absolute lymphocyte counts (median 546.8 vs 781.6 × 10^9^/L, *P* = 0.003) and higher concentrations of ferritin (1035 vs 529 ng/mL, *P* = 0.002) and lactate dehydrogenase (LDH; median 423 vs 339 U/L, *P* = 0.01) on the day of respiratory failure than did the patients without cancer. The patients with cancer also had higher mean arterial pressure scores (median 1 vs 0.5, *P* = 0.022) and a higher prevalence of vasopressor use (43.1% vs 28%, *P* = 0.03) on the day of respiratory failure than did the patients without cancer.

**Fig. 1:**
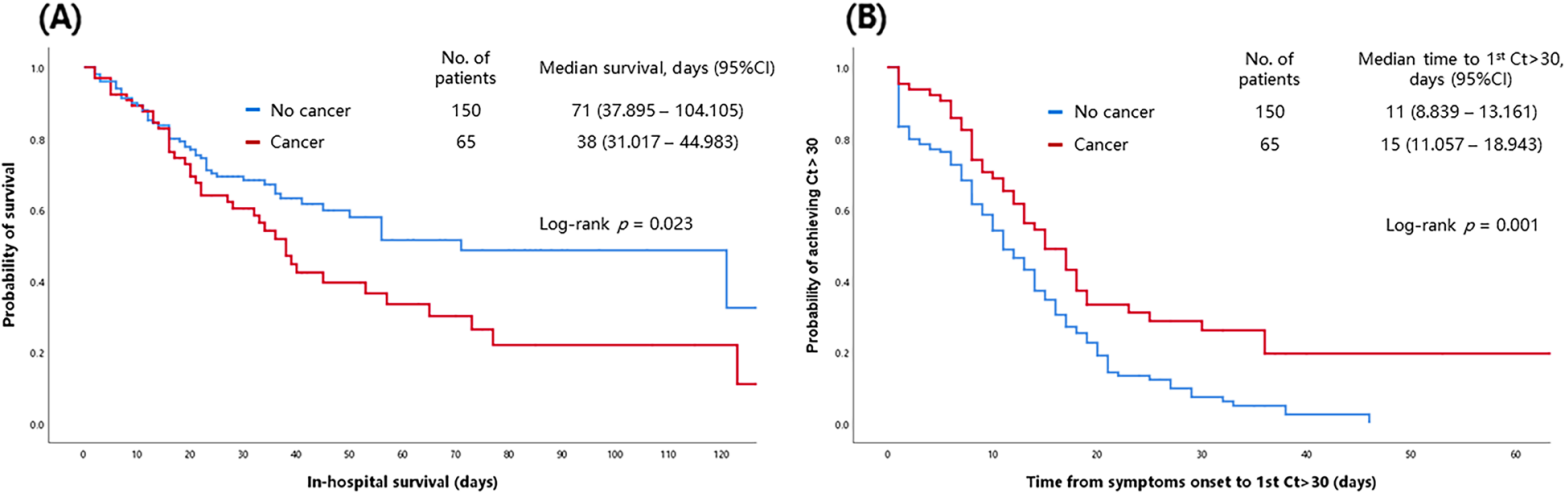
The in-hospital survival and time to reach Ct > 30 in patients with and without cancer. CI, confidence interval; Ct, cycle threshold

The patients with cancer were more likely to receive remdesivir (90.8% vs 73.3%, *P* = 0.004), tocilizumab (46.2% vs 30.7%, *P* = 0.029), and corticosteroids (93.8% vs 82%, *P* = 0.023) than were the patients without cancer. In terms of outcomes, the patients with cancer were significantly more likely to die in hospital (in-hospital mortality rate 61.5% vs 36%, *P* = 0.002) and took longer to reach Ct > 30 (median 13 vs 10 days, *P* = 0.007) than did the patients without cancer. The in-hospital survival and time to reach Ct > 30 in patients with and without cancer are illustrated in Fig. 1.

**Fig. 2:**
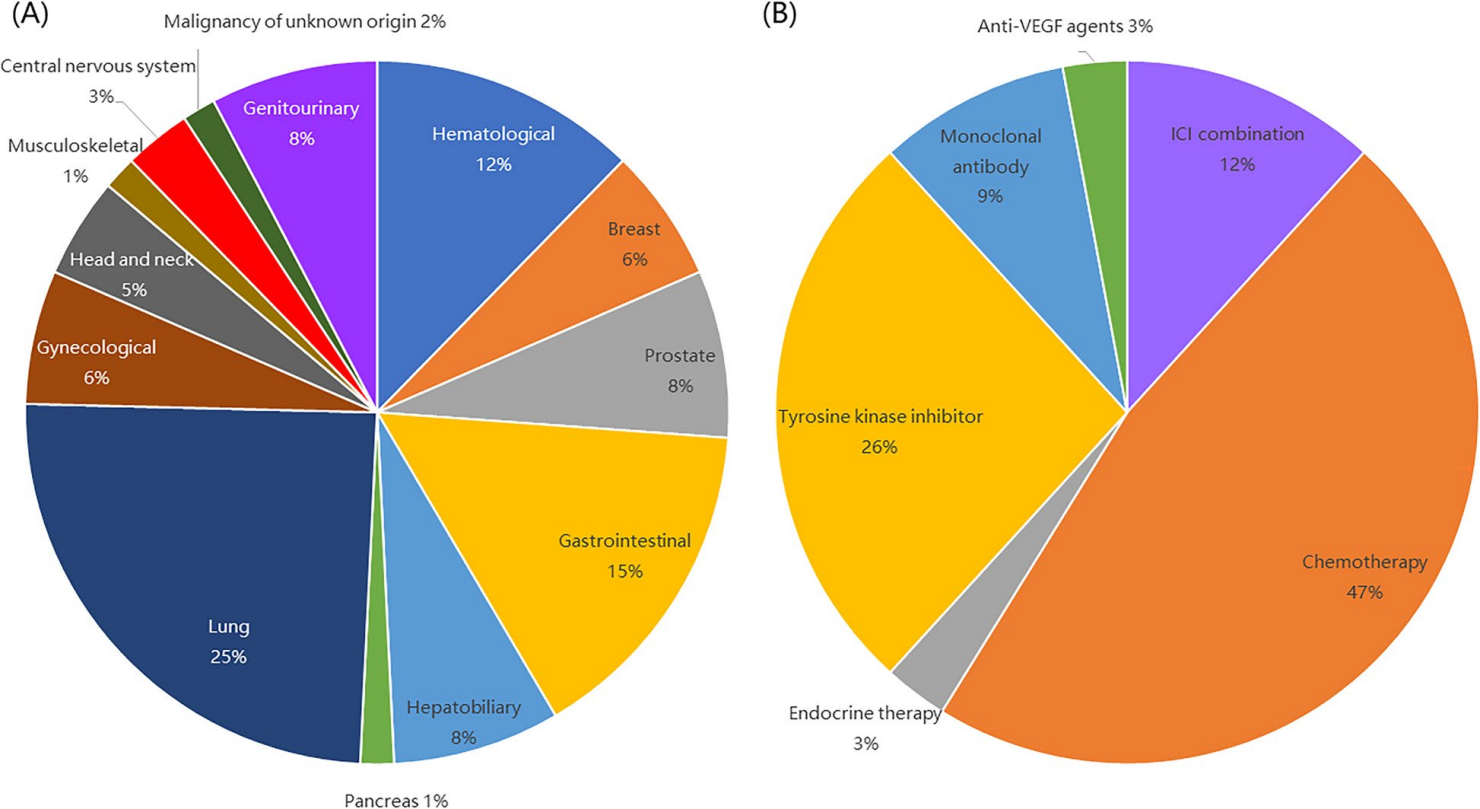
Cancer sites and recent treatment category among COVID-19 cancer patients with respiratory failure. VEGF, vascular endothelial growth factor

The characteristics of the 65 patients with cancer are summarized in Table 2 and Fig. 2.

**Table 2:** Additional characteristics among COVID-19 cancer patients with respiratory failure. (*n* = 65)

	*n* (%)
Cancer site	
Hematological	8 (12.3)
Lymphoma	7(10.8)
Myeloproliferative neoplasm	1(1.5)
Solid tumors	57 (87.7)
Breast	4(6.2)
Prostate	5(7.7)
Gastrointestinal	10(15.4)
Hepatocellular carcinoma	4(6.2)
Biliary tract	1(1.5)
Pancreas	1(1.5)
Lung	16(24.6)
Gynecological	4(6.2)
Head and neck	3(4.6)
Genitourinary	5(7.7)
Musculoskeletal	1(1.5)
Central nervous system	2(3.1)
Malignancy of unknown origin	1(1.5)
Cancer treatment within 4 weeks of COVID-19 diagnosis	34 (52.3)
ICI* combination	4 (6.2)
ICI + chemotherapy	3 (4.6)
ICI + TKI*	1(1.5)
Cytotoxic chemotherapy	16 (24.6)
Intravenous chemotherapy	12 (18.5)
Oral chemotherapy	4 (6.2)
Endocrine therapy	1 (1.5)
TKI	9 (13.8)
Monoclonal antibody	3 (4.6)
Anti-VEGF* agents	1 (1.5)

*ICI immune checkpoint inhibitor, *TKI* tyrosine kinase inhibitor, *VEGF* vascular endothelial growth factor

Most (87.7%) patients with cancer had solid tumors, with lung cancer (24.6%) and gastrointestinal tumors (15.4%) being the most common, followed by hematological malignancies (12.3%). In total, 34 (52.3%) patients received cancer-related treatment within 4 weeks before receiving a COVID-19 diagnosis, with approximately half receiving cytotoxic chemotherapy.

In-hospital mortality among the patients with cancer was 61.5%, with 25 survivors and 40 nonsurvivors (Table 3). The nonsurvivors were more likely to be smokers (42.5% vs 12%, *P* = 0.024) than were the survivors. Furthermore, the nonsurvivors had higher white blood cell counts (median 12,450 vs 8500 × 10^9^/L, *P* = 0.006) and concentrations of ferritin (median 3220 vs 673.5 ng/mL, *P* < 0.001), LDH (median 534.5 vs 256 U/L, *P* < 0.001), lactate (median 33 vs 15.7 mg/dL, *P* = 0.005), and D-dimer (median 4.605 vs 1.570 μg/mL, *P* = 0.007) than did the survivors. Additionally, the nonsurvivors had a higher incidence of vasopressor use on the day of respiratory failure (55% vs 24%, *P* = 0.014) and new renal replacement therapy during admission (22.5% vs 4%, *P* = 0.044) than did the survivors. The difference in survival status was not statistically significant based on whether patients had undergone systemic treatment or received cytotoxic chemotherapy within the 4 weeks preceding their COVID-19 diagnosis.

**Table 3:** Comparison of patient characteristics between COVID-19 survivors and non-survivors among patients with cancer during hospital stay. (*n* = 65)

	Survivor (*n* = 25)	Nonsurvivor (*n* = 40)	*P* value
**Demographics**			
Age, years, median	75	72.5	0.212
Male	13(52)	30(75)	0.057
Body mass index, kg/m^2^, median	21.04	23.17	0.229
BMI* < 18	7(28)	5(14.7)	0.210
BMI* > 24	9(36)	14(41.2)	0.687
Vaccination doses, median	2	2	0.890
Ever vaccinated	17(68)	28(70)	0.865
Full vaccination (> = 3 doses)	12(48)	12(48)	0.875
DNR*	15(60)	32(80)	0.080
Smoker	3(12)	17(42.5)	0.024
Cerebrovascular disease	4(16)	2(5)	0.194
Dementia	2(8)	3(7.5)	0.941
Heart failure	1(4)	0(0)	0.202
Peripheral vascular disease	1(4)	1(2.5)	0.733
Diabetes mellitus	9(36)	13(32.5)	0.772
Chronic kidney disease	3(12)	9(22.5)	0.288
End stage renal disease	1(4)	4(10)	0.377
Chronic obstructive pulmonary disease	2(8)	2(5)	0.624
Chronic oxygen use	2(8)	1(2.5)	0.304
Admitted due to COVID-19	12(48)	20(50)	0.875
Infected during hospitalization	3(12)	9(22.5)	0.344
Hematological malignancy	1(4)	6(15)	0.235
**Laboratory data on the day of respiratory failure (median)**			
White blood cells, 10^9^/L	8500	12,450	0.006
Absolute neutrophil count, 10^9^/L	6318.7	7105.45	0.345
Hemoglobin, g/dL	11.5	10.5	0.153
Absolute lymphocyte count, 10^9^/L	639.58	531.40	0.571
Albumin, g/dL	3.1	3.1	0.349
C-reactive protein, mg/dL	4.78	7.16	0.157
Procalcitonin, ng/mL	0.51	1.42	0.375
Ferritin, ng/mL	673.5	3220	< 0.001
Lactic dehydrogenase, U/L	256	534.5	< 0.001
Lactate, mg/dL	15.7	33	0.005
D-dimer, ug/mL	1.570	4.605	0.007
Fibrinogen, mg/dL	435.6	358	0.188
Platelet count, /uL	159,000	154,000	0.422
Severity on the day of respiratory failure			
PaO2/FiO2 ratio, median	148	125.39	0.364
SOFA* score, median	7	10	0.071
APACHE* II score, median	22	25.5	0.160
MAP score**, median	1	3	0.048
GCS*, median	9	7.5	0.995
Vasopressor use	6(24)	22(55)	0.014
**Treatment**			
Cancer treatment in 4 weeks prior to COVID-19 diagnosis	12(48)	22(55)	0.583
Cytotoxic chemotherapy in 4 weeks prior to COVID-19 diagnosis	8(32)	11(27.5)	0.698
Mechanical ventilation	18(72)	24(60)	0.325
Re-application of MV* after weaning	2(8)	2(5)	0.624
Tracheostomy	3(12)	1(2.5)	0.121
New renal replacement therapy during admission	1(4)	9(22.5)	0.044
Extracorporeal membrane oxygenation	0(0)	5(12.5)	0.066
Tocilizumab	9(36)	21(52.5)	0.194
Remdesivir	22(88)	37(92.5)	0.542
Nirmatrelvir/ritonavir	1(4)	3(7.5)	0.568
Molnupiravir	1(4)	1(2.5)	0.733
Enoxaparin	9(36)	13(32.5)	0.772
Corticosteroid	22(88)	39(97.5)	0.121
**Complications**			
CMV* infection	2(8)	14(35)	0.048
Gastrointestinal bleeding	6(24)	11(27.5)	0.755
Thromboembolism	3(12)	4(10)	0.800
**Outcome**			
ICU* admission	20(80)	28(70)	0.372
Hospital length of stay, days, median	33	21.5	0.082
Ventilator days, median	10	5.5	0.710
Time from symptoms onset to 1st Ct* > 30, days	14	11.5	0.721
Prolonged shredding (> 10 days)	18(72)	21(52.5)	0.118

*BMI Body mass index, *DNR* Do not resuscitate, *SOFA* Sequential Organ Failure Assessment, *APACHE* Acute Physiology and Chronic Health Evaluation, *MAP* Mean arterial pressure, *GCS* Glasgow coma scale, *CMV* Cytomegalovirus, *ICU* Intensive Care Unit, *Ct* cycle threshold

**MAP score is defined from the calculation of SOFA score, with inotropic doses as mcg/kg/min: 0, No hypotension; 1, MAP < 70 mmHg; 2, Dopamine ≤5 or Dobutamine (any dose); 3, Dopamine > 5, Epinephrine ≤0.1, or norepinephrine ≤0.1; 4, Dopamine > 15, Epinephrine > 0.1, or Norepinephrine > 0.1

The comparison of the characteristics, laboratory data, treatment, complications, and outcomes between cancer patients who have undergone recent systemic treatment and those who have not received it was summarized in Supplemental Table 1. The patients who have underwent cancer treatment were younger (median 71.5 vs 79 years old, *P* = 0.029), had lower absolute lymphocyte count on the day of respiratory failure (median 657.6 vs 440.28 × 109/L, *P* = 0.030), higher LDH level (median 536 vs 342 U/L, *P* = 0.016), and took shorter to reach Ct > 30 (median 8.5 vs 17 days, *P* = 0.033) than did the patients without treatment.

According to multivariable analysis (Table 4), smoking (OR: 5.804, 95% CI: 1.847–39.746, *P* = 0.043), an elevated concentration of LDH (OR: 1.004, 95% CI: 1.001–1.012, *P* = 0.025), vasopressor use on the day of respiratory failure (OR: 5.437, 95% CI: 1.202–24.593, *P* = 0.028), and new renal replacement therapy during admission (OR: 3.523, 95% CI: 1.203–61.108, *P* = 0.034) were significantly associated with in-hospital mortality among patients with cancer and COVID-19-related respiratory failure.

**Table 4:** Factors associated with in-hospital survival among COVID-19 cancer patients with respiratory failure (n = 65)

	Univariable analysis			Multivariable analysis		
Variables	Odds ratio	95% CI	*P* value	Odds ratio	95% CI	*P* value
Male	2.769	0.958–8.009	0.060	1.050	0.090–12.266	0.969
DNR	2.667	0.876–8.122	0.084	11.605	0.478–281.509	0.132
Smoker	5.420	1.392–21.107	0.015	5.804	1.847–39.746	0.043
Lactic dehydrogenase, U/L	1.004	1.001–1.007	0.008	1.004	1.001–1.012	0.025
Lactate, mg/dL	1.022	0.999–1.045	0.064	1.032	0.969–1.100	0.321
D-dimer, ug/mL	1.254	1.022–1.540	0.030	1.189	0.826–1.526	0.174
SOFA score	1.110	0.991–1.243	0.070	0.792	0.513–1.222	0.292
MAP score	1.415	1.028–1.948	0.033	0.330	0.037–2.936	0.320
Vasopressor use	3.870	1.276–11.735	0.017	5.437	1.202–24.593	0.028
New renal replacement therapy during admission	6.968	0.825–58.844	0.075	3.523	1.203–61.108	0.034
CMV infection	0.889	0.780–1.014	0.080	1.264	0.840–1.901	0.260

*CI* confidence interval, *DNR* do not resuscitate, *SOFA* sequential organ failure assessment, *MAP* mean arterial pressure, *CMV* cytomegalovirus

## Discussion

This study revealed the characteristics and factors that influence in-hospital mortality among patients with cancer and COVID-19-related respiratory failure during the period in which the omicron variant of SARS-CoV-2 was circulating in Taiwan. The patients with cancer and COVID-19-related respiratory failure exhibited distinct clinical characteristics, including lower lymphocyte counts, higher ferritin and LDH concentrations, and increased vasopressor use than did the patients without cancer. Additionally, the patients with cancer received COVID-19-related treatments more frequently than did the patients without cancer; however, in-hospital mortality was higher among the patients with cancer than among those without cancer. Smoking, an elevated LDH concentration, vasopressor use, and new renal replacement therapy were independent predictors of in-hospital mortality among this population.

The patients with cancer were generally younger and less likely to have histories of cerebrovascular accidents and heart failure than were the patients without cancer. This finding indicates that comorbidities other than advanced stage cancer contributed to the development of severe disease. The patients with cancer had lower absolute lymphocyte counts and higher ferritin and LDH concentrations on the day of respiratory failure than did the patients without cancer. Other biomarkers, such as C-reactive protein (CRP), lactate, fibrinogen, D-dimer, and procalcitonin, did not significantly differ between the patients with and without cancer. In Cai et al., among patients with COVID-19, those with cancer had higher concentrations of inflammatory markers and cytokines (high-sensitivity C-reactive protein, procalcitonin, interleukin (IL)-2 receptor, IL-6, and IL-8) and fewer immune cells than did those without cancer, indicating that patients with cancer are more susceptible to immune dysregulation [17]. Lymphopenia is a marker of COVID-19 severity and may be used to detect respiratory failure [20–22]. Patients with COVID-19 who are critically ill often exhibit hyperferritinemia; however, ferritin concentration is not a reliable predictor of patient outcomes [23–25]. An elevated LDH concentration has also been associated with mortality among patients with COVID-19 with severe disease and acute respiratory distress syndrome [22, 26–28].

In the present study, we discovered that the patients with cancer were more frequently treated with remdesivir, tocilizumab, and corticosteroids than were those without cancer. Use of enoxaparin and oral antivirals (nirmatrelvir/ritonavir and molnupiravir) did not significantly differ between the patients with and without cancer. Interleukin (IL)-6, known to be associated with adverse clinical outcomes in patients with COVID-19 [29], is also a key cytokine in the tumor microenvironment. IL-6, present in high concentrations in various cancer types, correlates with cancer progression and therapeutic resistance [30, 31]. IL-6 deregulation participates in the systemic hyperactivated immune response commonly referred to as the cytokine storm. Corticosteroids modulate inflammation-mediated lung injury and thereby reduce the likelihood of short-term mortality and the need for mechanical ventilation [6, 32]. Tocilizumab, a monoclonal antibody against IL-6 receptor, reduces the likelihood of progression to mechanical ventilation or death in patients hospitalized with COVID-19 and is effective among patients with COVID-19 with various cancer types [33–35]. We propose that corticosteroids and tocilizumab were used more frequently among the patients with cancer than among those without cancer due to the hyperinflammatory status of the patients with cancer, whose inflammatory status was confirmed by their elevated concentrations of inflammatory markers (ferritin and LDH). The immunocompromised status of the patients with cancer may have led to active viral replication; therefore, although remdesivir was used more frequently among the patients with cancer than among those without cancer, the patients with cancer took longer to reach Ct > 30. The patients with cancer exhibited prolonged nasopharyngeal viral RNA shedding. Longer viral shedding is associated with older age, distant metastasis, and more severe COVID-19 disease [36].

The patients with cancer had higher MAP scores and a greater likelihood of vasopressor use on the day of respiratory failure than did those without cancer, indicating greater hemodynamic instability among these patients. The patients with cancer were also demonstrated a significantly higher in-hospital mortality rate compared to those without cancer, which is consistent with the finding of another study [37].

Among the patients with cancer in our study, in-hospital mortality was associated with smoking; a higher white blood cell count; and elevated concentrations of ferritin, LDH, lactate, and D-dimer. These factors indicate that an active inflammatory process may have contributed to a poor prognosis. The nonsurvivors with cancer were also significantly more likely to use vasopressors and receive new renal replacement therapy during their admission than were the survivors.

Vaccination status, comorbidities, recent systemic cancer treatment, whether admitted due to COVID-19, whether infected during hospitalization, SOFA score and APACHE II score on the day of respiratory failure, and specific treatments for COVID-19 (including those involving corticosteroids, antiviral and anticoagulation agents, and tocilizumab) did not significantly affect mortality.

In multivariable analysis, we identified several factors that were associated with in-hospital mortality among the patients with cancer and COVID-19-related respiratory failure. These factors included smoking, elevated LDH concentrations on the day of respiratory failure, requiring vasopressor use on the day of respiratory failure, and undergoing new renal replacement therapy during admission.

Active smoking is considered as an independent predictor of severe disease and mortality among patients with COVID-19 [9, 38–40]. Current smokers had significantly increased ACE2 expression in airway epithelial cells compared with nonsmokers, which provided more entry points for the SARS-CoV-2 virus and potentially increased susceptibility to infection [41]. However, in one study, active smoking was not associated with COVID-19 severity [42]. Elevated LDH concentration has been identified as an independent risk factor for disease severity and mortality among patients with COVID-19 [27, 28, 43]. The requirement for mechanical ventilation, vasopressors, and renal replacement therapy were reported to be poor prognostic factors among patients with cancer who were admitted to the intensive care unit [44]. Patients with COVID-19 who are admitted to the intensive care unit frequently receive continuous vasopressor support [45], highlighting the importance of hemodynamic monitoring and fluid management.

Our study has several limitations. First, this was a single-center retrospective cohort study with a limited sample size. Second, the laboratory data and SARS-CoV-2 PCR follow-up intervals were not uniform, which potentially introduced bias. Third, some inflammatory biomarkers such as IL-6, IL-2R, IL-8 and antibody titers are either not routinely tested or have no available exam in our hospital, thus we do not have sufficient data to incorporate into our analysis. Treatment strategies may have also varied considerably by patient clinical status and clinician practice.

## Conclusion

Patients with cancer who develop COVID-19-related respiratory failure exhibit distinct clinical characteristics and have a higher likelihood of receiving specific COVID-19 treatments, such as remdesivir and corticosteroids, than those without cancer do. Patients who develop COVID-19-related respiratory failure with cancer also experience unfavorable outcomes, including higher in-hospital mortality and longer duration of viral shedding, compared with those without cancer. Smoking, elevated LDH concentrations, vasopressor use, and new renal replacement therapy were identified as significant predictors of in-hospital mortality in this patient population. Further research is warranted to validate these findings, elucidate the underlying mechanisms, and explore tailored management strategies to improve outcomes in this vulnerable population.

### Supplementary Information

**Additional file 1.**

## Data Availability

The datasets used and/or analyzed during the current study available from the corresponding author on reasonable request.

## Abbreviations

COVID-19: Coronavirus disease 2019
LDH: Lactate dehydrogenase
OR: Odds ratio
CI: Confidence interval
ARDS: Acute respiratory distress syndrome
SARS-CoV-2: Severe acute respiratory syndrome coronavirus 2
HFNC: High-flow nasal cannula
NIV: Noninvasive ventilation
MV: Mechanical ventilation
RT-PCR: Reverse transcription polymerase chain reaction
BMI: Body mass index
DNR: Do not resuscitate
SOFA: Sequential organ failure assessment
MAP: Mean arterial pressure
APACHE: Acute Physiologic Assessment and Chronic Health Evaluation
CMV: Cytomegalovirus
Ct: Cycle threshold
IL: Interleukin
