# Supplementary material for: Clinical characteristics and outcomes among critically ill patients with cancer and COVID-19-related acute respiratory failure
Source: BMC Pulm Med. 2024 Jan 15;24:34. doi: 10.1186/s12890-024-02850-z (PMC10789018; doi:10.1186/s12890-024-02850-z)
Supplement: Supplementary file 1 — Additional file 1. [file 12890_2024_2850_MOESM1_ESM.docx]

Supplementary Table 1. Comparison of patient characteristics between patients who had and who had not received cancer treatment in 4 weeks prior to COVID-19 diagnosis. (n=65)

|  | Cancer treatment in 4 weeks prior to COVID-19 diagnosis (n=34) | No cancer treatment in 4 weeks prior to COVID-19 diagnosis (n=31) | *P* value |
| --- | --- | --- | --- |
| **Demographics** | | | |
| Age, years, median | 71.5 | 79 | 0.029 |
| Male | 9 (26.5) | 25 (80.6) | 0.188 |
| Body mass index, kg/m^2^, median | 23.04 | 22.44 | 0.308 |
| Smoker | 12 (35.3) | 8 (25.8) | 0.332 |
| Cerebrovascular disease | 1 (2.9) | 5 (16.1) | 0.095 |
| Dementia | 1 (2.9) | 4 (12.9) | 0.184 |
| Heart failure | 1 (2.9) | 0 (0) | 0.523 |
| Peripheral vascular disease | 0 (0) | 2 (6.5) | 0.224 |
| Diabetes mellitus | 8 (23.5) | 14 (45.2) | 0.066 |
| Chronic kidney disease | 4 (11.8) | 8 (25.8) | 0.145 |
| End stage renal disease | 3 (8.8) | 2 (6.5) | 0.720 |
| Chronic obstructive pulmonary disease | 1 (2.9) | 3 (9.7) | 0.341 |
| Chronic oxygen use | 1 (2.9) | 2 (6.5) | 0.602 |
| **Laboratory data on the day of respiratory failure (median)** | | | |
| White blood cells, 10^9^/L | 9430 | 11340 | 0.485 |
| Hemoglobin, g/dL | 10.75 | 10.7 | 0.470 |
| Absolute neutrophil count, 10^9^/L | 6544.95 | 6548.5 | 0.415 |
| Absolute lymphocyte count, 10^9^/L | 440.28 | 657.6 | 0.030 |
| Albumin, g/dL | 3.1 | 2.95 | 0.955 |
| C-reactive protein, mg/dL | 9.29 | 5.46 | 0.237 |
| Procalcitonin, ng/mL | 1.05 | 0.99 | 0.949 |
| Ferritin, ng/mL | 3220 | 888.5 | 0.100 |
| Lactic dehydrogenase, U/L | 536 | 342 | 0.016 |
| Lactate, mg/dL | 26.4 | 32.96 | 0.496 |
| D-dimer, ug/mL | 3.89 | 2.29 | 0.287 |
| Fibrinogen, mg/dL | 391.15 | 377.8 | 0.655 |
| Platelet count, /uL | 122000 | 164000 | 0.090 |
| Severity on the day of respiratory failure | | | |
| PaO2/FiO2 ratio, median | 142 | 133.94 | 0.946 |
| SOFA* score, median | 8 | 9 | 0.756 |
| APACHE* II score, median | 25 | 24 | 0.828 |
| MAP* score, median | 1 | 1 | 0.680 |
| GCS*, median | 10 | 7 | 0.465 |
| Vasopressor use | 13 (38.2) | 15 (48.4) | 0.409 |
| **Treatment** | | | |
| Mechanical ventilation | 19 (55.9) | 23 (74.2) | 0.123 |
| Re-application of MV* after weaning | 2 (5.9) | 2 (6.5) | 0.924 |
| Tracheostomy | 2 (5.9) | 2 (6.5) | 0.924 |
| New renal replacement therapy during admission | 4 (11.8) | 6 (19.4) | 0.500 |
| Extracorporeal membrane oxygenation | 2 (5.9) | 3 (9.7) | 0.663 |
| Tocilizumab | 14 (41.2) | 16 (51.6) | 0.399 |
| Remdesivir | 30 (88.2) | 29 (93.5) | 0.674 |
| Nirmatrelvir/ritonavir | 2 (5.9) | 2 (6.5) | 0.924 |
| Molnupiravir | 1 (2.9) | 1 (3.2) | 0.947 |
| Enoxaparin | 10 (29.4) | 12 (38.7) | 0.429 |
| Corticosteroid | 33 (97.1) | 28 (90.3) | 0.259 |
| **Complications** | | | |
| CMV* infection | 9 (26.5) | 7 (22.6) | 0.469 |
| Gastrointestinal bleeding | 9 (26.5) | 8 (25.8) | 0.951 |
| Thromboembolism | 4 (11.8) | 3 (9.7) | 0.786 |
| **Outcome** | | | |
| ICU* admission | 23 (67.6) | 25 (80.6) | 0.234 |
| Hospital length of stay, days, median | 22.5 | 33 | 0.318 |
| Ventilator days, median | 3 | 13 | 0.097 |
| Time from symptoms onset to 1^st^ Ct* >30, days | 8.5 | 17 | 0.033 |
| Prolonged shredding (>10 days) | 16 (47.1) | 23 (74.2) | 0.026 |

*BMI, Body mass index; SOFA, Sequential Organ Failure Assessment; APACHE, Acute Physiology and Chronic Health Evaluation; MAP, Mean arterial pressure; GCS, Glasgow coma scale; CMV, Cytomegalovirus; ICU, Intensive Care Unit; Ct, cycle threshold

**MAP score is defined from the calculation of SOFA score, with inotropic doses as mcg/kg/min: 0, No hypotension; 1, MAP <70 mmHg; 2, Dopamine ≤5 or Dobutamine (any dose); 3, Dopamine >5, Epinephrine ≤0.1, or norepinephrine ≤0.1; 4, Dopamine >15, Epinephrine >0.1, or Norepinephrine >0.1
